# Multiple polypoid lesions with erosion of the gastric mucosa in adult T‐cell lymphoma/leukemia superimposed on cytomegalovirus infection

**DOI:** 10.1002/jha2.476

**Published:** 2022-06-24

**Authors:** Yutaka Shimazu, Fumihiko Kouno, Keisuke Shindo, Shinsaku Imashuku, Takashi Miyoshi

**Affiliations:** ^1^ Department of Hematology Uji Tokushukai Medical Center Uji Japan; ^2^ Department of Pathology Uji Tokushukai Medical Center Uji Japan

**Keywords:** cytomegalovirus, HTLV‐1, malignant lymphoma

**Abbreviations**
ATLL: adult T‐cell leukemia/lymphoma
CMV: cytomegalovirus

1

We report here unique endoscopic findings of cytomegalovirus (CMV) gastritis associated with adult T‐cell leukemia/lymphoma (ATLL). A 67‐year‐old Japanese man presented to a hematologist with a month history of appetite loss. Physical examination showed blurred consciousness (GCS E4V2M5) with labored breathing and hypotension. Blood examination revealed hypercalcemia (adjusted Ca: 3.24 mmol/L), anemia (Hb: 70 g/L), and leukocytosis (white blood cell count: 21.3 × 10^9^/L) with abnormal lymphocytes (7%). Human T‐cell lymphotropic virus type 1 antibody and provirus DNA monoclonality were positive. A blood transfusion was given, and noradrenaline and denosumab were administered. Upper gastrointestinal tract endoscopy showed multiple polypoid lesions with redness and erosive surface (upper left; see Figure 1). A gastric‐biopsy specimen showed the presence of CD3^+^CD4^+^CD8^−^ T cells, compatible with the gastric involvement by ATLL, and CMV gastritis presenting with inclusion bodies positive for anti‐CMV antibody by immunohistochemistry (upper right and lower left; see Figure 1). CMV antigenemia was present, and soluble interleukin‐2 receptor was markedly elevated at 119,194 U/ml. CMV enteritis was also observed. He was treated with ganciclovir for CMV gastroenteritis. One‐month after repeated endoscopy revealed a significant regression of redness and erosion of polypoid lesions (lower right; see Figure 1). Two months later, he has been undergoing chemotherapy for ATLL. Since gastric involvement by ATLL presenting as multiple lymphomatous polyposis could only been observed in advanced stage [1, 2], we seldom encounter with this lesion nowadays. Although CMV antigenemia is frequently observed in ATLL patients [3], we rarely treat ATLL patients with CMV infections, particularly gastritis. The present case illustrates the rare complication of ATLL in these days (see Figure 1).

**FIGURE 1:**
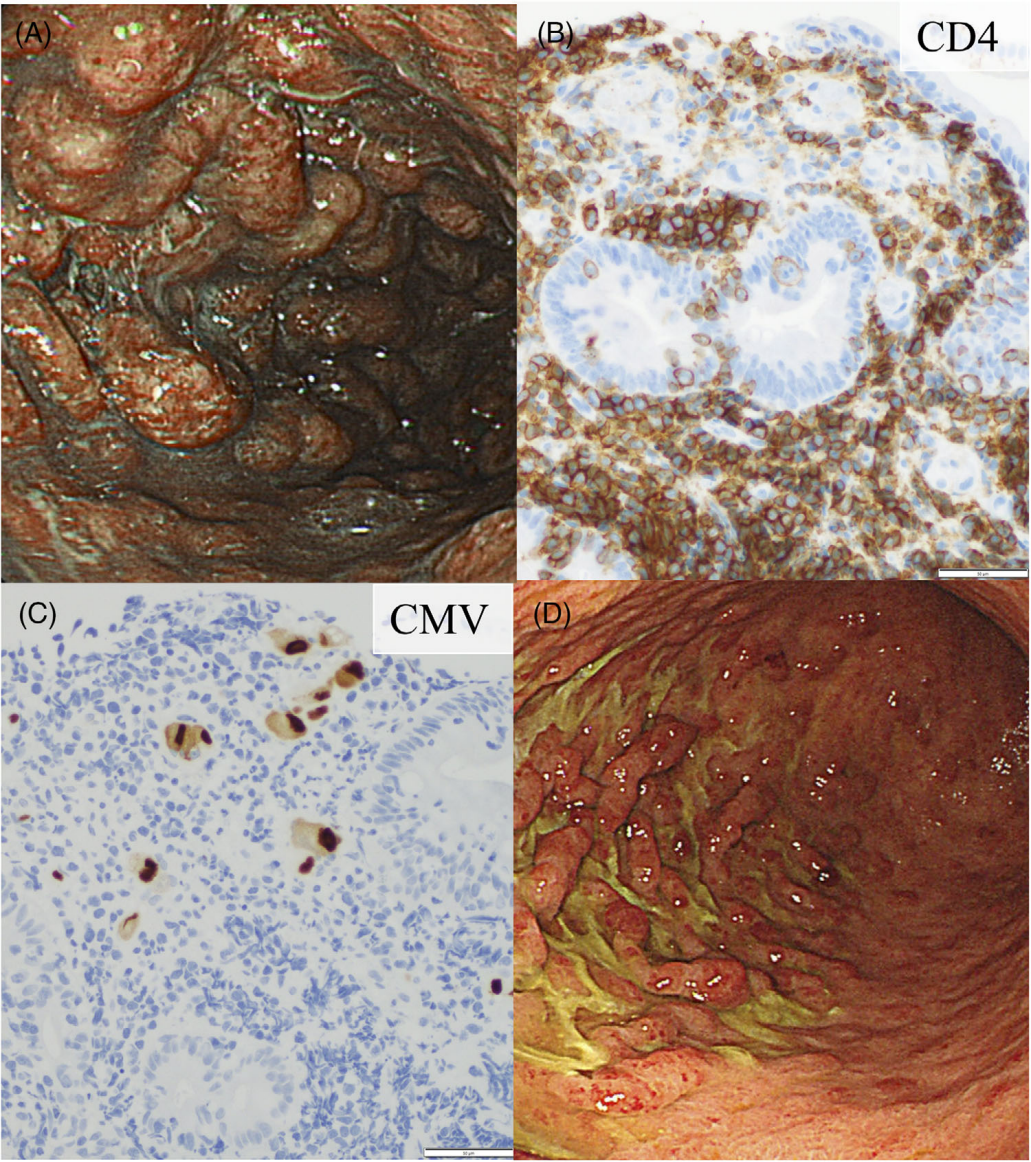
Photographs of upper gastrointestinal tract endoscopy at diagnosis (A). Immunohistochemistry (magnification ×200) of anti‐CD4 staining (B) and cytomegalovirus (CMV) antigen (C). The photographs of upper gastrointestinal tract endoscopy after treatment (D)

## CONFLICT OF INTEREST

All the authors have no conflict of interest.

## FUNDING INFORMATION

The authors received no specific funding for this work.

## ETHICS STATEMENT

All procedures performed in this study involving the patient were in accordance with the ethical standards of our institutional and national research committee and with the 1964 Helsinki declaration and its later amendments or comparable ethical standards.

## AUTHOR CONTRIBUTIONS

Conceptualization, Methodology, Investigation, Data curation, Visualization, Writing‐Reviewing and Editing: Yutaka Shimazu. Investigation, Data curation and Visualization: Fumihiko Kouno. Investigation, Writing‐Reviewing and Editing: Shinsaku Imashuku. Investigation, Writing‐Reviewing and Editing: Keisuke Shindo. Investigation, Supervision and Writing‐Reviewing and Editing: Takashi Miyoshi.
